# Supplementary material for: Microvalve-Based Tunability of Electrically Driven Ion Transport through a Microfluidic System with an Ion-Exchange Membrane
Source: Anal Chem. 2023 Apr 11;95(16):6514–22. doi: 10.1021/acs.analchem.2c04600 (PMC10134142; doi:10.1021/acs.analchem.2c04600)
Supplement: Supplementary file 1 — ac2c04600_si_001.pdf [file ac2c04600_si_001.pdf]

Supplementary Materials

Microvalve-Based Tunability of Electrically Driven Ion Transport Through a Microfluidic System with Ion-Exchange Membrane

Barak Sabbagh[1], Sinwook Park[2], Gilad Yossifon[1,2*]

[1]*Faculty of Mechanical Engineering, Technion–Israel Institute of Technology, Israel*

[2]*School of Mechanical Engineering, Tel-Aviv University, Israel*

* Corresponding author: gyossifon@tauex.tau.ac.il

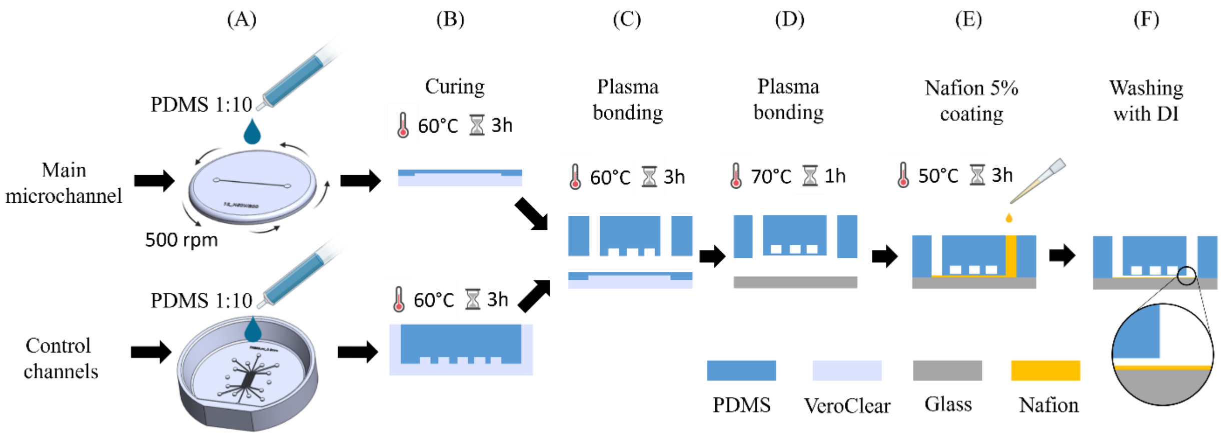

Figure S1: Schematic of the fabrication process of the microfluidic chip. (A) Degassed 10:1 polydimethylsiloxane (PDMS, base: crosslinker, Sylgard 184) was spin-coated (for the main microchannel) or casted (for the control channels) onto 3D-printed molds (PolyJet technology, material VeroClear, Synergy ©). Spin-coating (500rpm, 20sec) was used to ensure a thin PDMS layer (~60µm) above the main microchannel structure. (B) PDMS curing at 60°C () over 3h. (C) Peeling of the cured PDMS from the control channel mold, punching holes (2mm diameter) for inlets and outlets, and bonding it on top of the cured PDMS of the main channel using manual oxygen plasma (BD-20A generator, Electro-Technic Products). The control channels were placed orthogonally to the main channel. For effective bonding, the chip was incubated at 60 °C for 3h. (D) Peeling of the cured main channel (including the bonded control channels on top), punching inlets holes (1.5mm diameter), and plasma bonding (following the same protocol as before) of the bottom surface of the main channel to a glass slide. (E) Introduction of Nafion solution (Nafion™ 1100W, Sigma-Aldrich) through one of the inlets of the main microchannel and curing at 50 °C for 3h. Due to poor adhesion between the PDMS and the Nafion, it is mostly the glass slides that are coated. The coating thickness (2 ± 1 µm) is approximated by the water percentage of Nafion solution and the main microchannel geometry (i.e., ~50µm height). (F) Washing the main microchannel using deionized (DI) water.

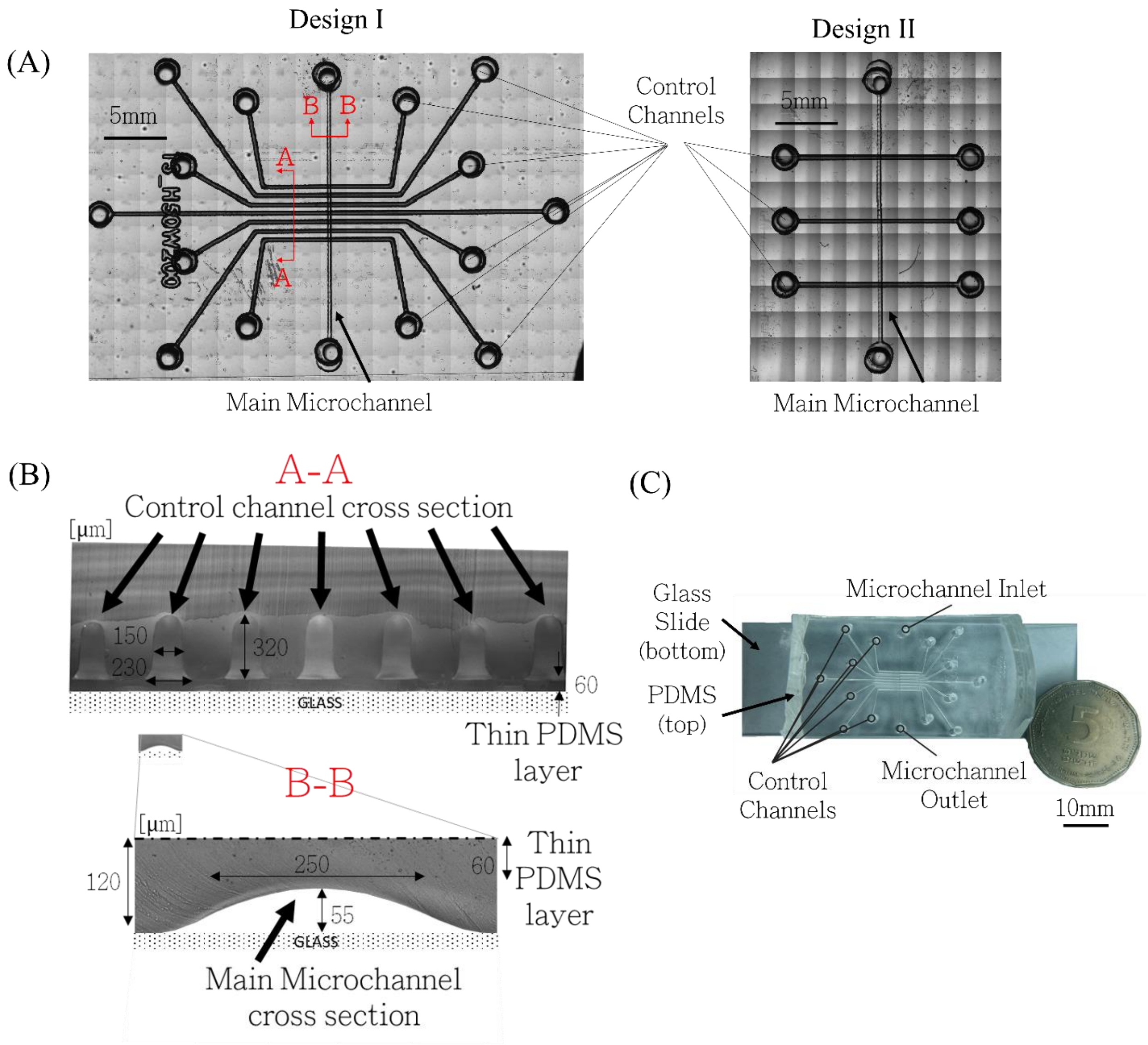

Figure S2: Microscopic images of microfluidic chip. (A) Photo montage of images obtained via an automated x-y stage scan of the two chip designs described in this paper. The main differences between the designs were the number of control channels (i.e., number of microvalves), and the gap between the control channels. Design (I): 6 control channels with a 300µm separation gap. Design (II): 3 control channels with a 5mm separation gap. (B) Cross-sectional images (design (II)): Top image – the top PDMS layer containing 7 control channels, bonded to an intermediate PDMS thin layer (main microchannel is not seen in the cut). Bottom image – the PDMS layer that contains the main microchannel. (C) An image of the assembled chip (design (II)) in proportion to an Israeli 5-shekel coin.

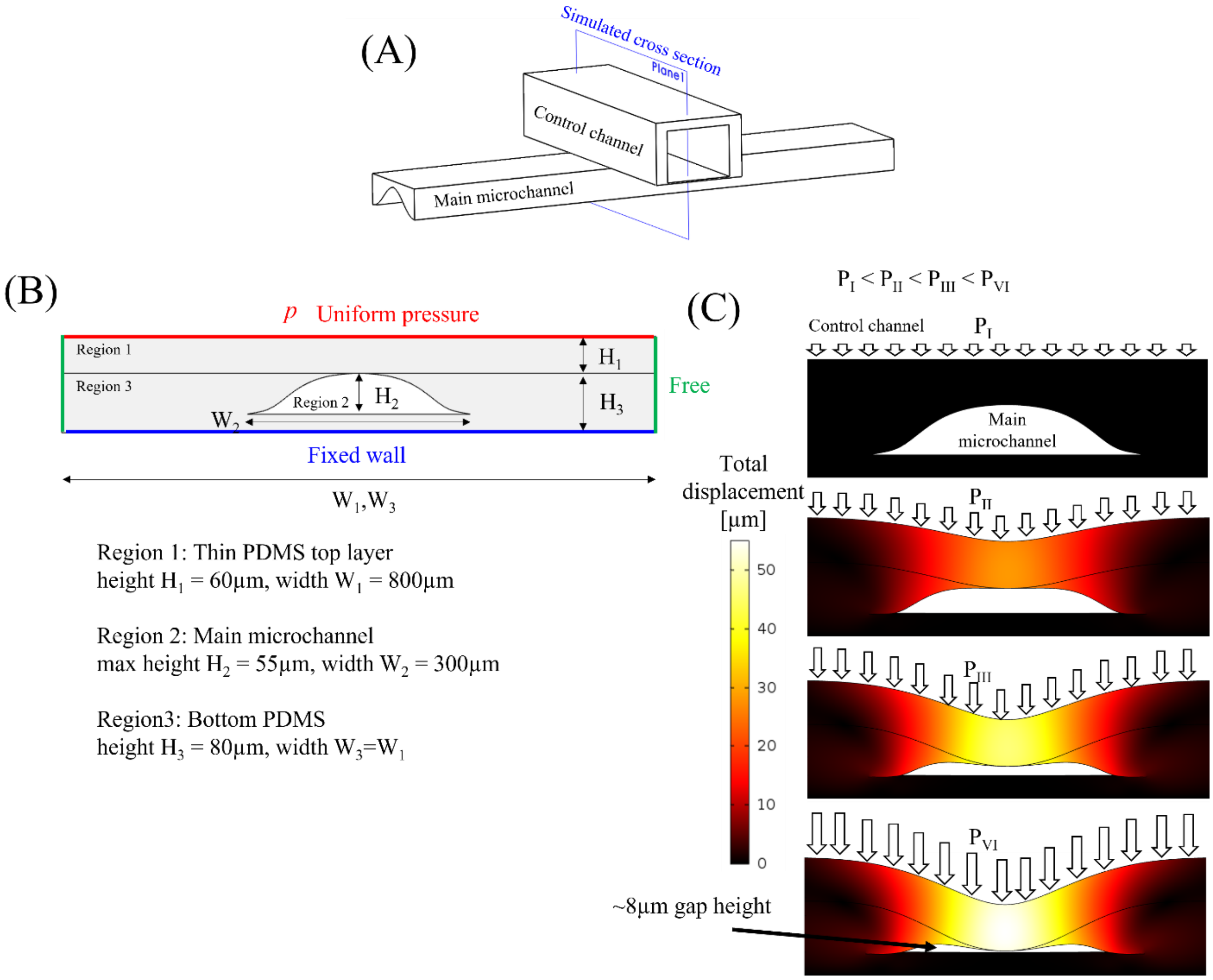

Figure S3: **Numerical simulation (COMSOL Multiphysics, solid mechanics) of the microvalve deformation.** (A) Schematic of the simulated two-dimensional central cross-section of the chip. (B) The defined geometry for each region of the simulated cross-section. The main microchannel shape was determined based on Fig. S2B, where the boundary conditions used were: fixed wall (blue line), free wall (green line), uniformly distributed P (red line). (C) The deformations obtained under various pressure values (P_I<P_{II}<P_{III}<P_{VI}, represented by the white arrows).

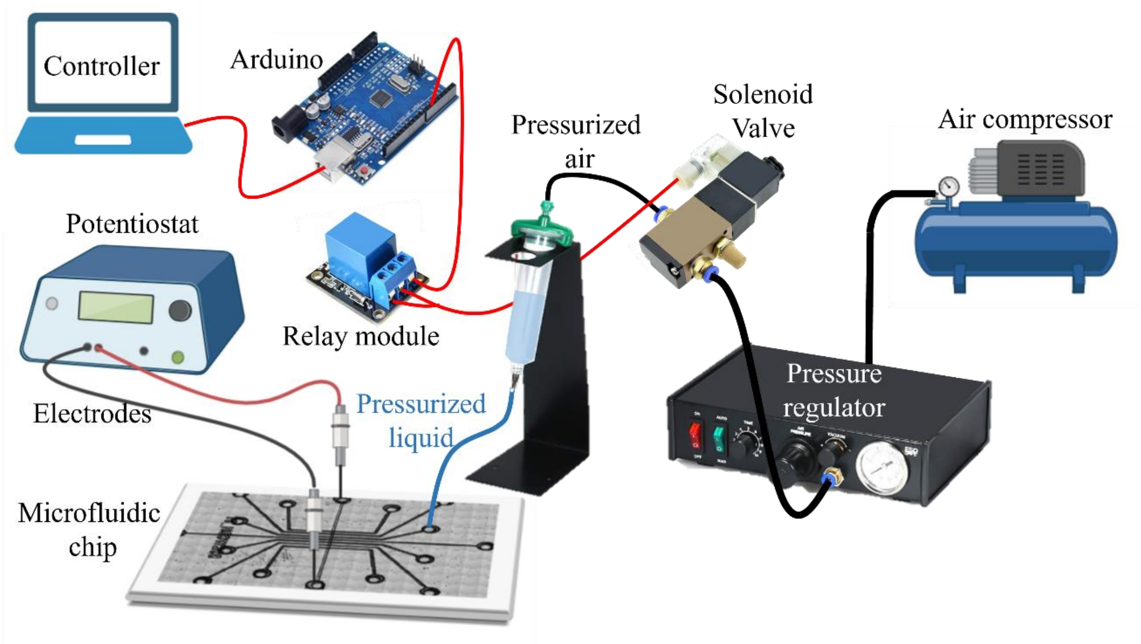

Figure S4: **Schematic of the study's experimental setup.** The home-made pressure control system consisted of an air compressor connected to a pressure regulator (220V AD-982 Semi-Auto Glue Dispenser) with an output pressure P. Air (black line) was pressure-converted to liquid (blue line) within a sealed tube (20ml), where a pressurized air enters from above and the pressurized liquid (DI water) exits from below through a tubing to the microchannel inlet. We have used pressurized liquid[S1–S3] instead of air within the control channel to eliminate the possible gas permeation[S4–S6] across the thin membrane layer and the introduction of undesired gas bubbles within the main channel. A pneumatic valve (2V025-08 NC, ERICC) in between the pressure regulator and the tube, is connected to turn on (P) and off (atmospheric pressure) the pressurized air within the tube. A relay module (JQC-3FF-S-Z, TONGLING) wired to Arduino (Arduino Uno, Digi-Key®) was used to control the pneumatic valve. After washing the control channel with liquid, its outlet was temporary sealed. Each control channel (microvalve) was controlled by its own pneumatic valve, relay module, and tubing. Additional information on the experimental setup can be found in the MATERIALS AND METHODS section. Figure created with BioRender.com.

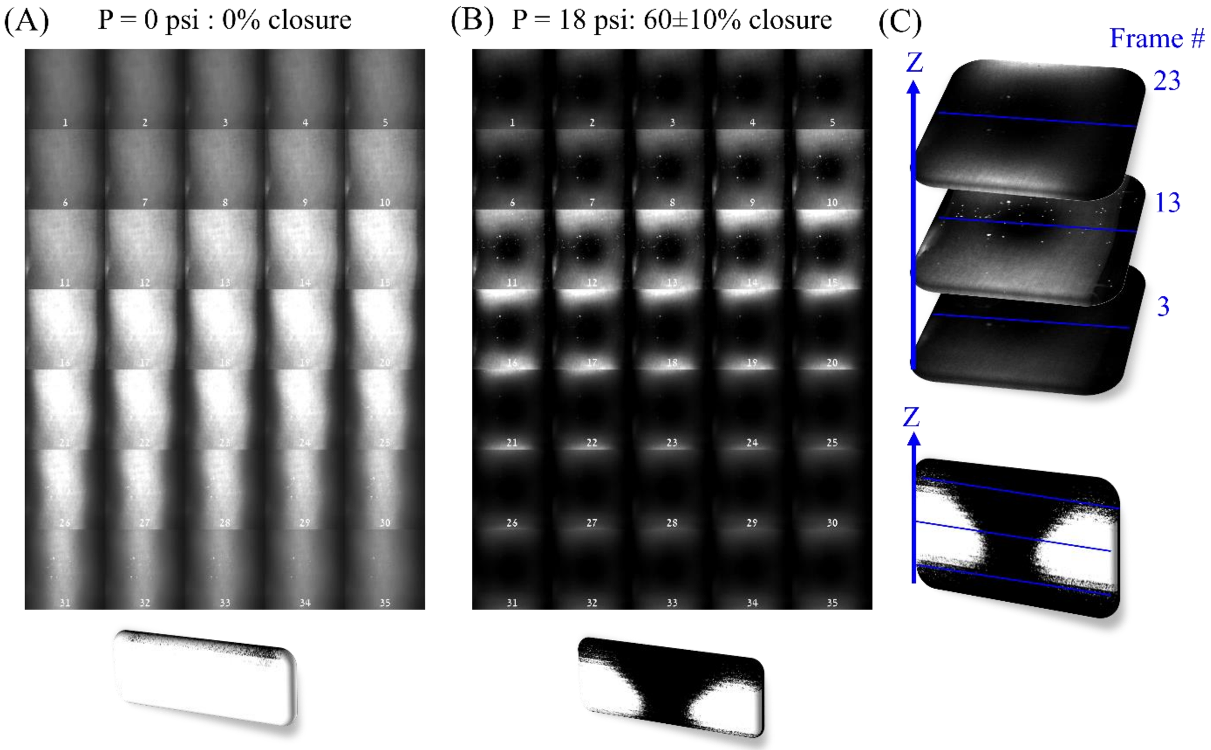

Figure S5: 3D reconstruction of the main microchannel underneath the microvalve. Confocal microscopy was used to visualize microvalve deformation. The 3D channel shape was reconstructed based on 2D images scanned on different focal planes (68 µm in 35 planes) by using confocal microscopy and a fluorescent dye (Dylight) which filled the main microchannel (control channel was filled with DI water). (A) Captured 2D images for deactivated microvalve with P=0psi. White color (high fluorescent intensity) represents an open volume (contains fluorescent solution), while black color (low fluorescent intensity) represents a close volume (solid PDMS). (B) Captured 2D imaged for activated microvalve with P=18psi. (C) 3D reconstruction of three images taken from (B). On the bottom, the cross section of the main channel after plotting all images (total 35) and showing only the intensity obtained at the center of each image (blue line).

Numerical simulations:

$$(S1)\ \partial c_i / \partial t = -\nabla j_i = D_i \nabla^2 c_i + z_i D_i F \nabla c_i \nabla^2 \phi / RT - U \nabla c_i, \quad i = +,\ -,\ A$$

$$(S2)\ \nabla^2 \phi = \rho_e / \varepsilon_0 \varepsilon_r$$

$$(S3)\ \mu \nabla^2 U = \nabla p, \quad U = (\text{ v,w })$$

$\partial c_i / \partial n = 0$ No ion penetration

$U = 0$ No slip velocity & No penetration

$\partial \phi / \partial n = 0$ Electrical isolation

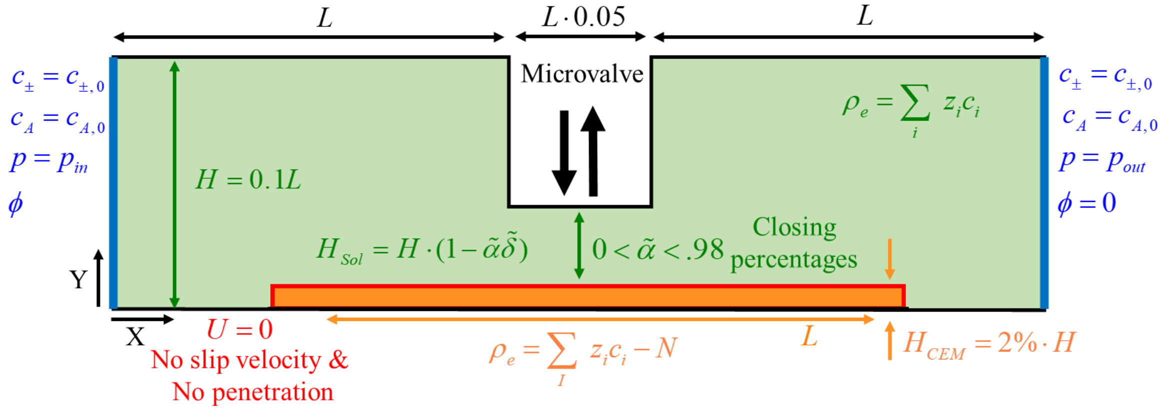

Figure S6: Numerical simulations model of our microvalve and Nafion coated microchannel system. The dimensional model's governing equations are: (S1) Nernst-Planck, (S2) Poisson, and (S3) Navier-Stokes. Herein, ρ_e is the ionic charge density, ε_0 and ε_r are the permittivity of the vacuum and the relative permittivity of the electrolyte, respectively, p is the hydrodynamic pressure, μ is the dynamic viscosity, and U is the velocity vector (wherein, v and w are the velocity components in x and y directions, respectively). Schematic of the simulated system geometry and boundary conditions at the inlets (left and right blue lines), microchannel inner walls (black), and CEM inner walls (red). $\partial / \partial n$ is the normal derivative. Green represents the electrolyte solution within the microchannel, while orange represents the CEM. The channel height below the microvalve (H_{Sol}, marked in grey) was defined as a function of the open microchannel height (H), an arbitrary variable $\tilde{\alpha}$ that varies between 0 and 0.98 (2% and 100% closure when taking the CEM height into account, respectively), and Kronecker's delta ($\tilde{\delta}(t - t_0)$), which was used to simulate dynamic microvalve closure at t_0.

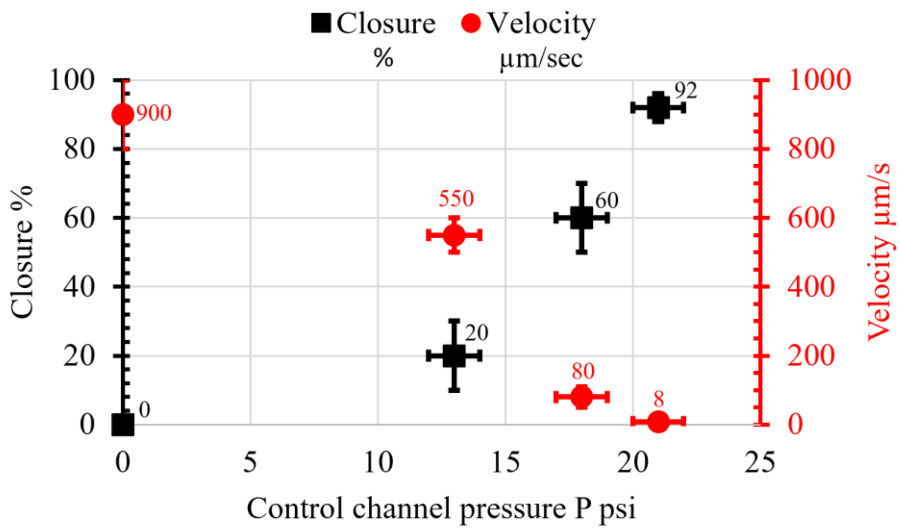

Figure S7: **The relation between the applied control channel pressure P (psi) to the main channel closure percentage (%) and the flow velocity (µm/s).** The closure percentage (black square, left y-axis) was approximated based on confocal imaging-based three-dimensional reconstruction of the cross-section shape of the main microchannel (Fig.S5). The velocity (red circle, right y-axis) was measured by monitoring 1µm polystyrene (Fig.S8).

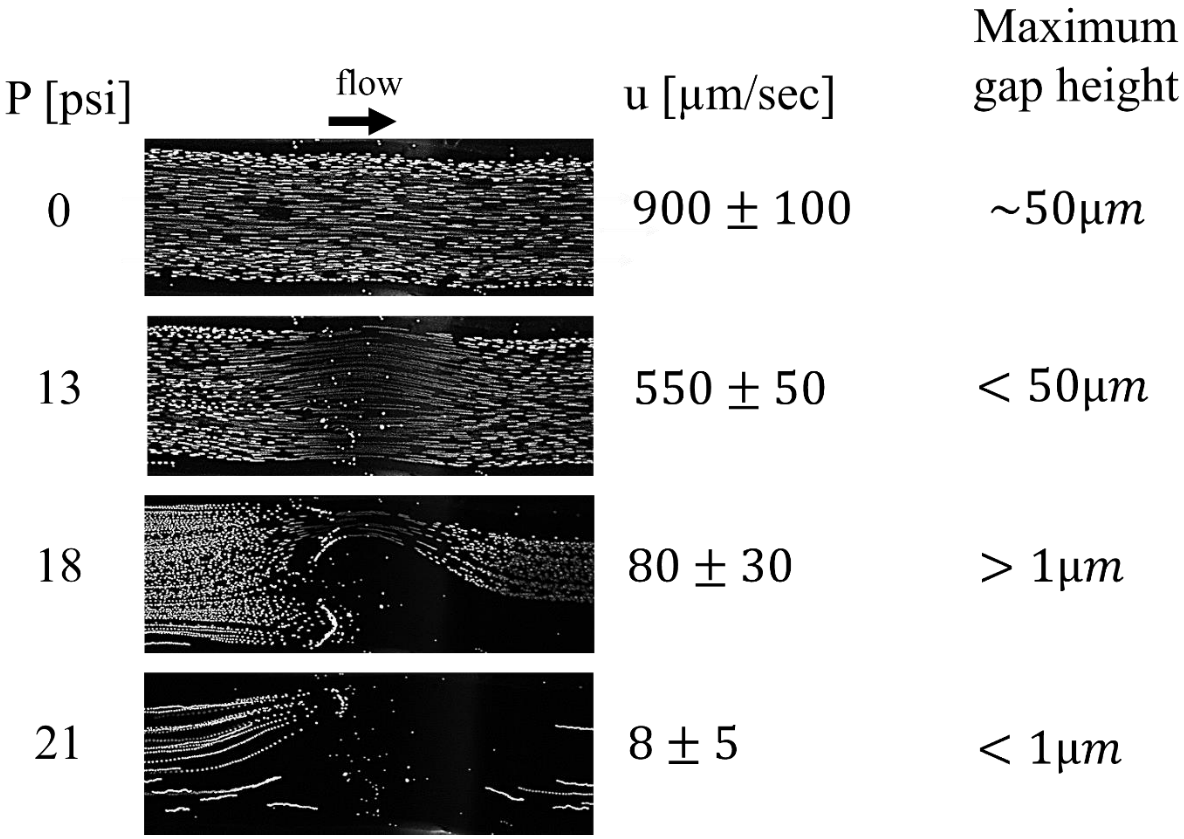

Figure S8: Particle trajectories and calculated average flow velocity for various control channel pressures (P=0,13,18,21psi). The trajectories of the particles (1µm polystyrene green fluorescent particles) were obtained by superimposing the fluorescence signals of these tracers over 100 frames (~0.1 frame/sec for P=0,13psi, and 1 frame/sec for all other conditions). The average velocity (u) for each P was calculated based on velocity measurements of 10 particles outside the microvalve region. At P=21psi, the particles could not pass through the microvalve region, while there was still flow of the solution, and therefore, the average gap was estimated to be less than 1µm.

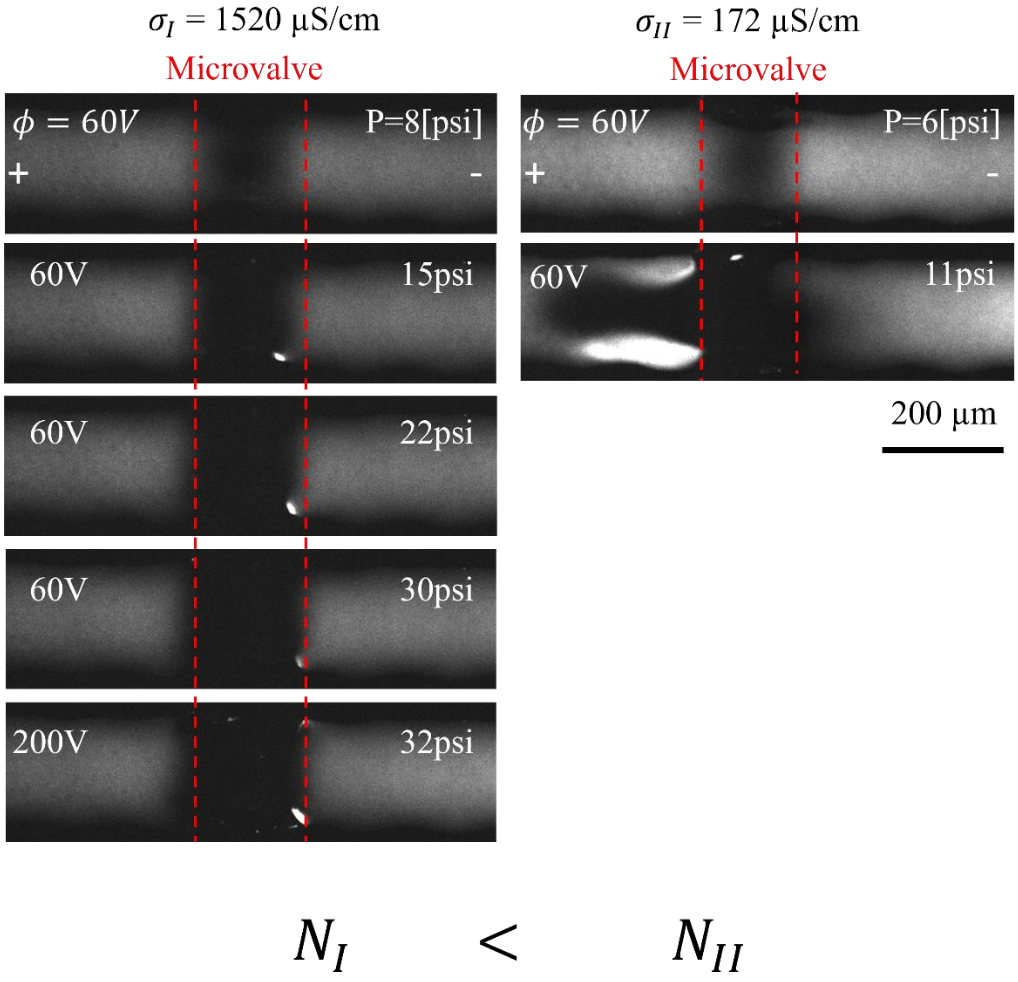

Figure S9: Experimental system response without a Nafion coating. With high electrolyte concentration (left column, ~10mM KCl, 1520 µS/cm), no ICP was obtained even for a relatively high P (32psi) and ϕ (200V). With a one-order-of-magnitude-lower electrolyte concentration (right column, ~1mM, 172 µS/cm), a weak ICP was obtained upon application of control channel pressure of 11psi and application of a voltage drop of 60V. This trend arises from the increased thickness of the electric double layer with decreases the electrolyte concentration, which, in turn, leads to an increase in ion-permselectivity of the formed nanochannels (e.g., $N_I < N_{II}$).

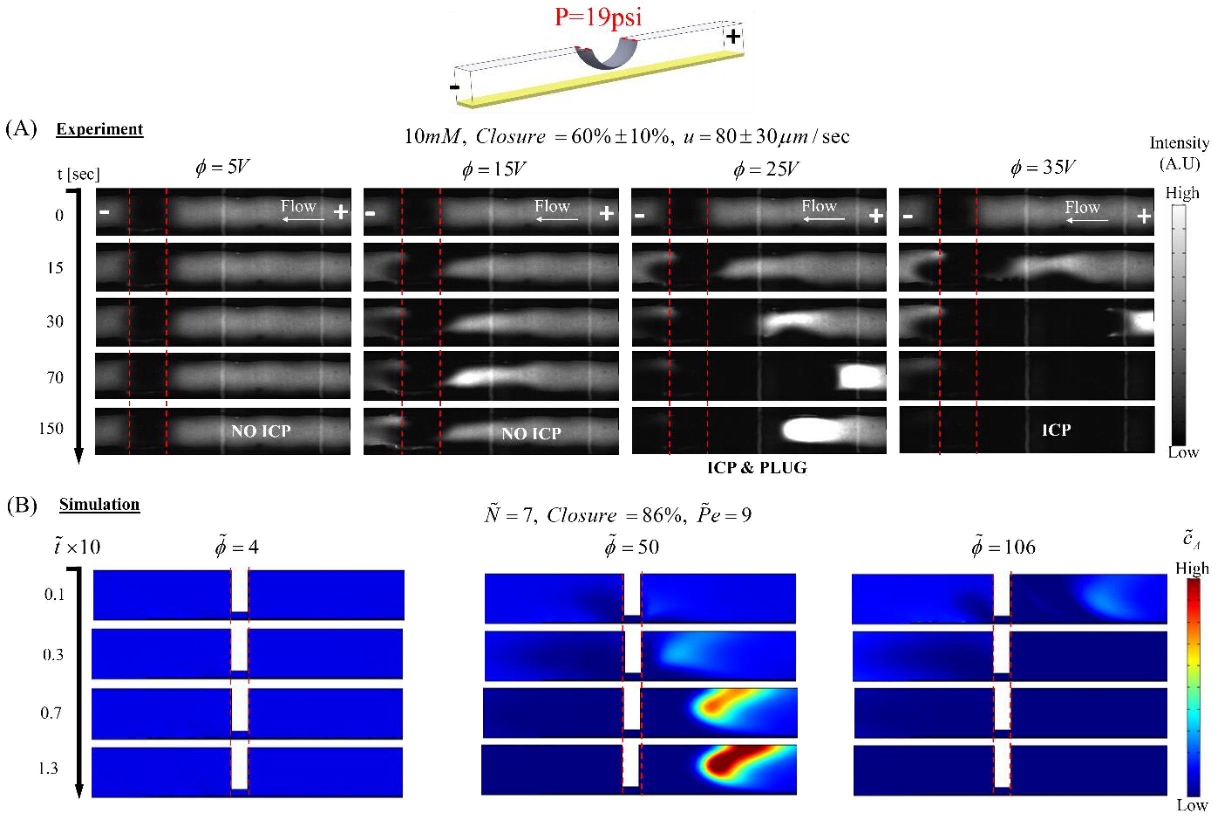

Figure S10: The effect of a constant microvalve deformation level on the system response to various voltage drops. (A) Experimental results showing the fluorescence intensity over time (0<t<150s) for different voltage drops ($\phi = 5,\ 15,\ 25,\ 35\text{V}$) and a constant pressure, P=19psi. (B) Calculated analyte concentration over time for different voltage drops ($\tilde{\phi} = 4,\ 50,\ 106$) at 86% main channel closure.

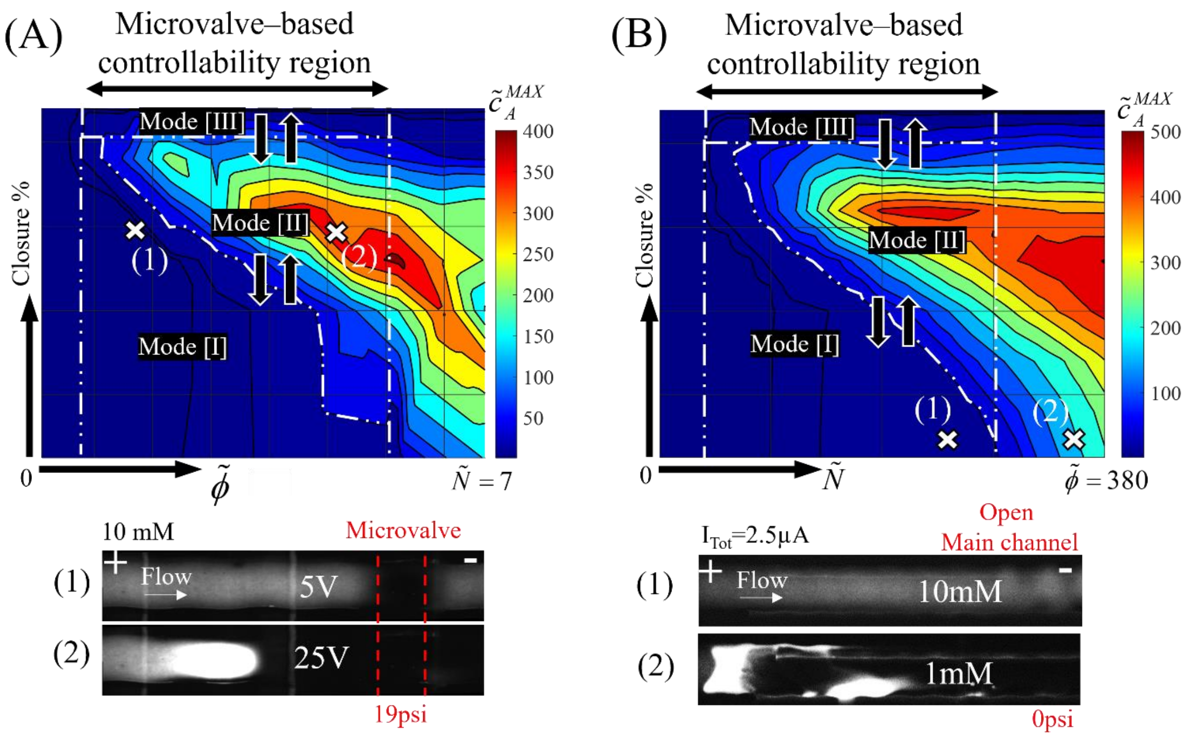

Figure S11: Limitations and optimization of microvalve-based controllability of ion concentration-polarization (ICP) driven preconcentrated analyte plug. Numerical simulation of the system response as shown in Fig.4, presenting the maximum analyte concentration ($\tilde{c}_A^{MAX}$) obtained at given time of $\tilde{t} = 10^{-2}$ within the main channel as a function of (A) $\tilde{\phi}$ (examined range of $0<\tilde{\phi}<700$, $\tilde{N}=7$, $\tilde{u}=65$) or (B) $\tilde{N}$ ($\tilde{\phi}=380$, $0<\tilde{N}<20$, $\tilde{u}=65$) for various closure percentages (0-96%). Below each phase diagram, two experimentally examined cases that qualitatively represent a similar response to the simulation are depicted. The selected cases for demonstrating the system response in different modes are marked with X (1) and (2) on the phase diagram.

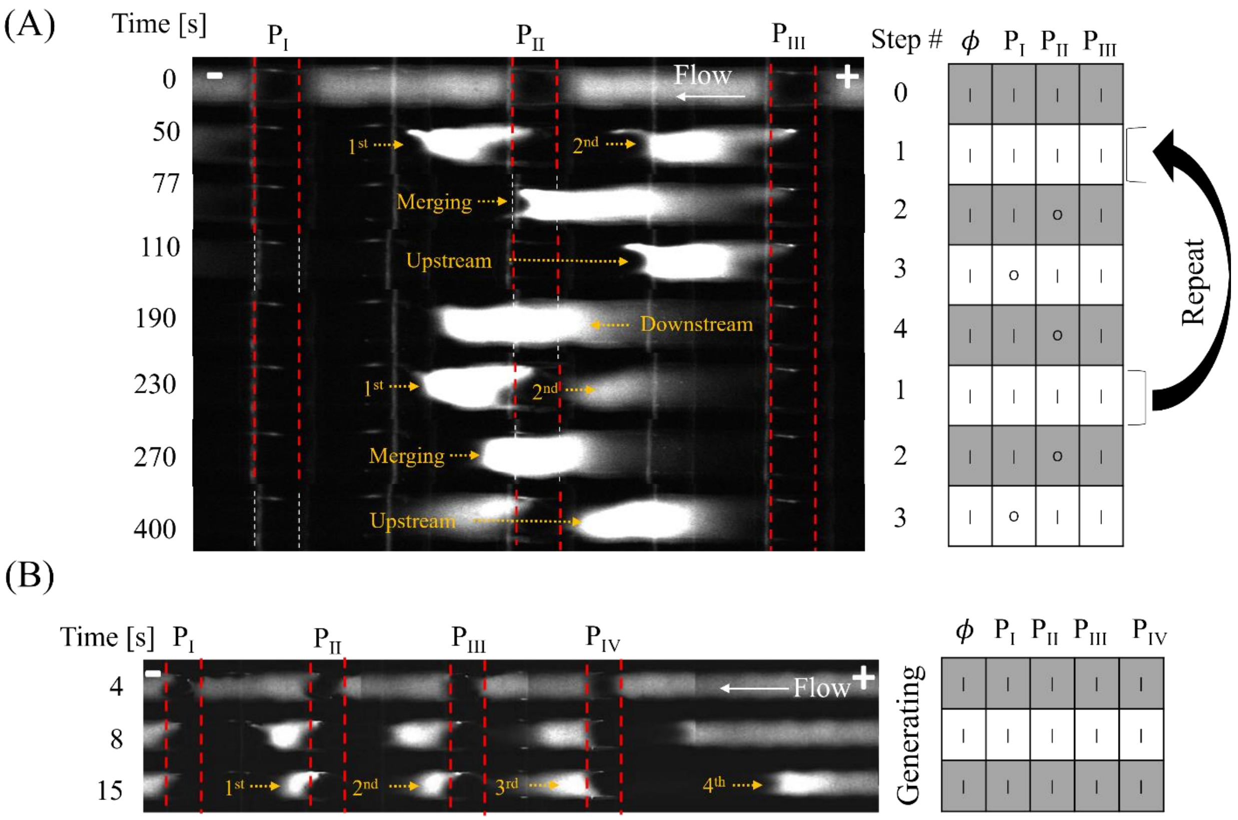

Figure S12: Dynamic operation of multiple plugs. (A) Down- and upstream translocation of a biomolecule plug by performing a sequence of microvalves operation: generation of two plugs (step 1), plug merging (step 2), upstream translation (step 3), downstream translation and repetition of the sequence (step 4). (B) Simultaneous generation of four plugs by activating four microvalves in series.

Movies

Movie M1, Experimental and numerical demonstration of on-demand ICP-based preconcentration plug formation by dynamic microvalve activation (AVI)

Movie M2, Experimental results of the effect of microvalve deformation level on the system response, including ICP generation, preconcentrated plug and solution flow (AVI)

Movie M3**,** Experimental demonstration of preconcentrated plug manipulation and trapping using multiple microvalves (AVI)

References

(S1) Tahvildari, R.; Beamish, E.; Briggs, K.; Chagnon-Lessard, S.; Sohi, A. N.; Han, S.; Watts, B.; Tabard-Cossa, V.; Godin, M. Manipulating Electrical and Fluidic Access in Integrated Nanopore-Microfluidic Arrays Using Microvalves. *Small* **2017**, *13* (10). https://doi.org/10.1002/smll.201602601.

(S2) Quist, J.; Trietsch, S. J.; Vulto, P.; Hankemeier, T. Elastomeric Microvalves as Tunable Nanochannels for Concentration Polarization. *Lab Chip* **2013**, *13*, 4810. https://doi.org/10.1039/c3lc50658d.

(S3) Sabbagh, B.; Stolovicki, E.; Park, S.; Weitz, D. A.; Yossifon, G. Tunable Nanochannels Connected in Series for Dynamic Control of Multiple Concentration-Polarization Layers and Preconcentrated Molecule Plugs. *Nano Lett.* **2020**, *20*, 8524–8533. https://doi.org/10.1021/acs.nanolett.0c02973.

(S4) Lamberti, A.; Marasso, S. L.; Cocuzza, M. PDMS Membranes with Tunable Gas Permeability for Microfluidic Applications. *RSC Adv.* **2014**, *4* (106), 61415–61419. https://doi.org/10.1039/c4ra12934b.

(S5) Johnson, M.; Liddiard, G.; Eddings, M.; Gale, B. Bubble Inclusion and Removal Using PDMS Membrane-Based Gas Permeation for Applications in Pumping, Valving and Mixing in Microfluidic Devices. *J. Micromechanics Microengineering* **2009**, *19* (9). https://doi.org/10.1088/0960-1317/19/9/095011.

(S6) Eddings, M. A.; Gale, B. K. A PDMS-Based Gas Permeation Pump for on-Chip Fluid Handling in Microfluidic Devices. *J. Micromechanics Microengineering* **2006**, *16* (11), 2396–2402. https://doi.org/10.1088/0960-1317/16/11/021.
